# A Rare Location of Polymorphous Low-Grade Neuroepithelial Tumor of the Young (PLNTY) of the Thalamus: A Case Report and Review of Literature

**DOI:** 10.7759/cureus.91224

**Published:** 2025-08-29

**Authors:** Ashwin Sridhar, Bhaskar Naidu P, Lawrence D'Cruze

**Affiliations:** 1 Neurological Surgery, Sri Ramachandra Institute of Higher Education and Research, Chennai, IND; 2 Pathology, Sri Ramachandra Institute of Higher Education and Research, Chennai, IND

**Keywords:** braf mutation, dnet, glioneuronal tumour, plnty, thalamus

## Abstract

Polymorphous low-grade neuroepithelial tumor of the young (PLNTY) is a rare CNS WHO Grade I brain tumor that typically affects children and young adults and may be associated with seizures. Thalamic involvement is exceedingly rare. We report a case of a 29-year-old male with persistent paresthesias in the right upper and lower limbs and suspected sensory seizures. Imaging revealed a left thalamic space-occupying lesion. Stereotactic biopsy confirmed PLNTY. The case highlights the rare presentation of PLNTY in an unusual location and in an adult, emphasising the need for accurate pathological diagnosis in atypical presentations.

## Introduction

Polymorphous low-grade neuroepithelial tumor of the young (PLNTY) is a rare, low-grade neuroepithelial tumor, first described by Huse et al. in 2017 [1], characterized by oligodendroglioma-like histology, CD34 positivity, and MAPK pathway mutations. It was incorporated into the 2021 WHO CNS classification under pediatric low-grade gliomas and glioneuronal tumors [2]. PLNTY typically arises in the temporal lobe, presenting with seizures in children and young adults [3,4]. Thalamic localization is rare and, to the best of our knowledge, not documented in the literature [5,6].

## Case presentation

A 29-year-old right-handed male presented to the outpatient department with a two-month history of numbness and paresthesia in the right upper limb, progressing to the lower limb. The symptoms were insidious in onset and progressive. There was no history of headache, visual disturbances, motor weakness, or trauma. However, a suspected episode of sensory seizure was reported by the caregiver. The patient had previously undergone a non-contrast CT brain at another hospital, which showed a left thalamic space-occupying lesion.

**Figure 1:**
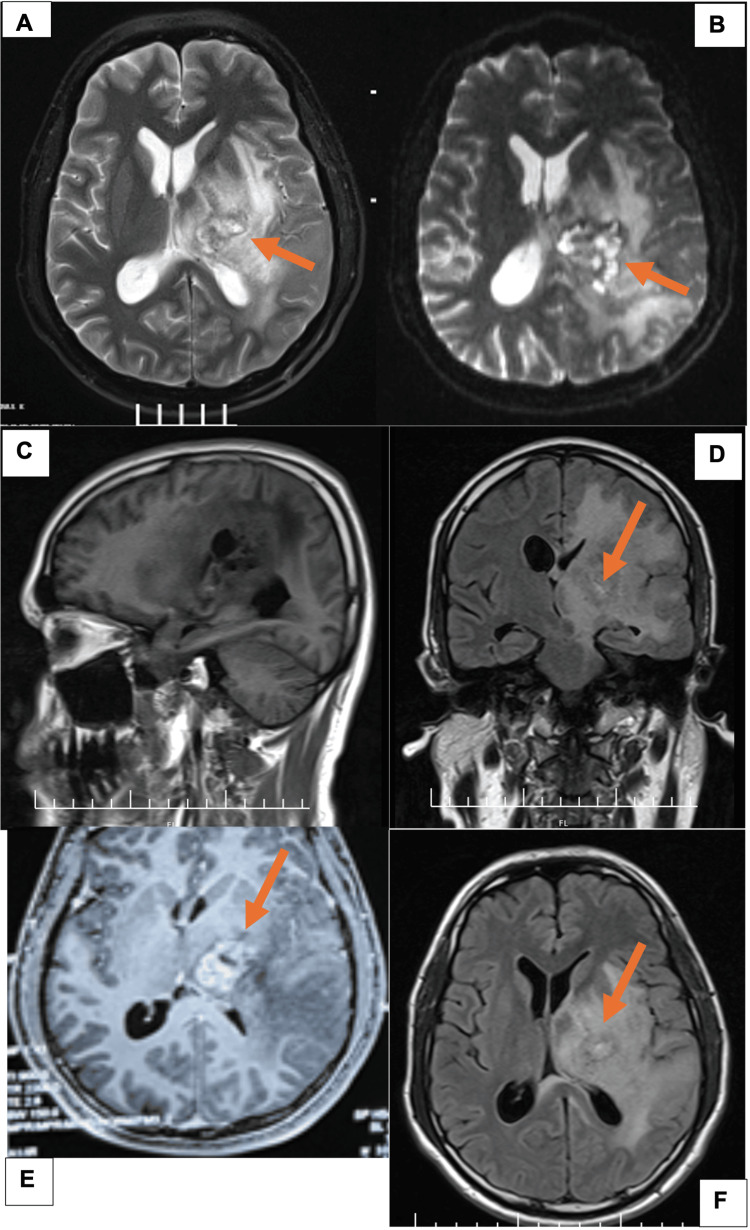
Preoperative MRI imaging Preoperative T2-weighted axial images (A & B), T1-weighted sagittal image (C), fluid-attenuated inversion recovery (FLAIR) coronal image (D), T1 post-contrast axial image (E), and FLAIR axial image (F).

On examination, the patient was alert and oriented. Neurological examination revealed minimal paresthesia in the right limbs but no focal motor or cranial nerve deficits. MRI brain with contrast demonstrated a solitary lesion in the left thalamus (Figure 1). The lesion was hypointense on T1-weighted imaging and hyperintense on T2-weighted imaging. It also showed no diffusion restriction and was hyperintense on fluid-attenuated inversion recovery (FLAIR) sequences with a partial enhancement on contrast administration. Susceptibility weighted imaging (SWI) revealed no hemorrhage or calcification.

**Figure 2:**
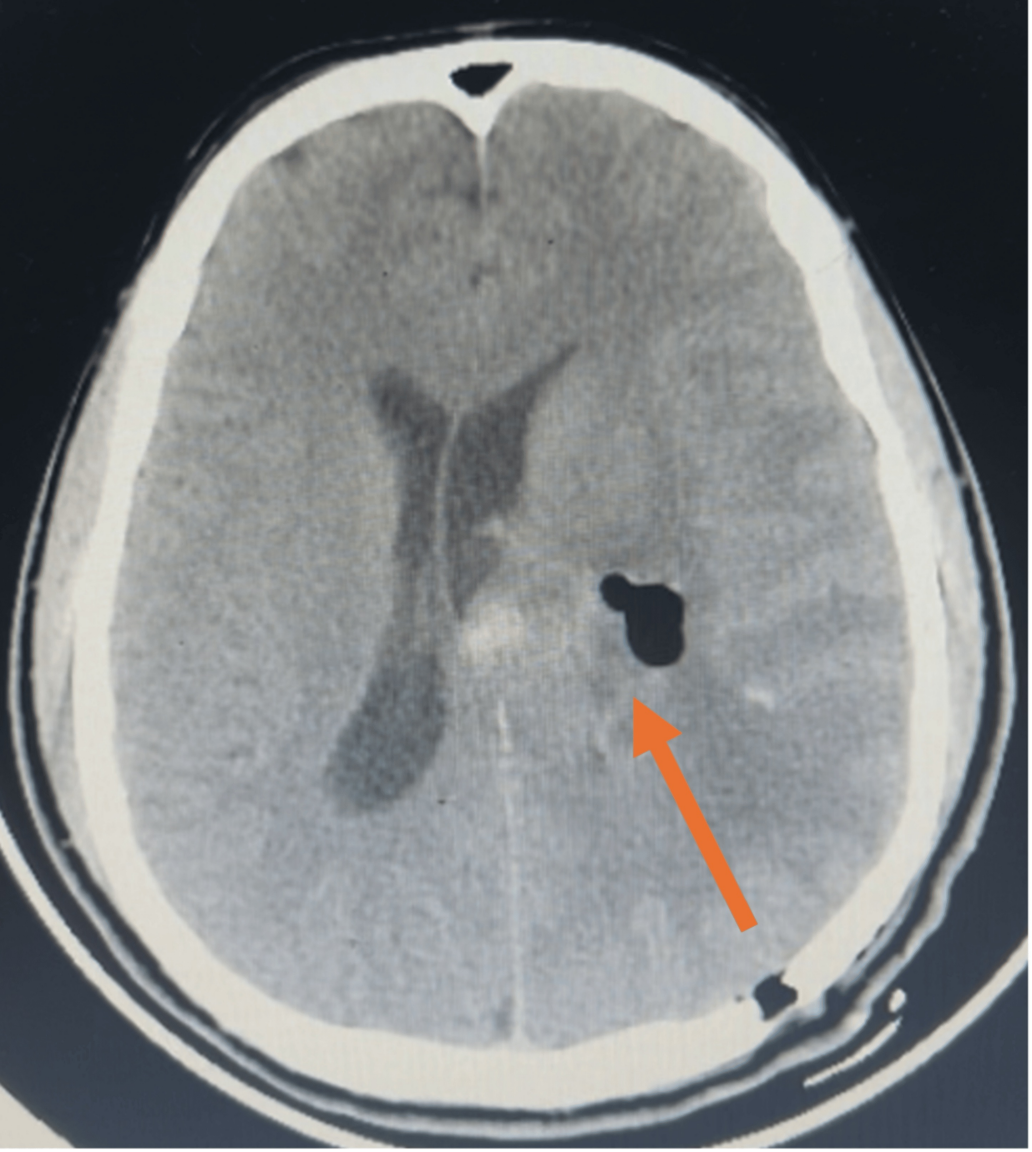
Postoperative non-contrast CT brain Postoperative CT brain plain highlighting the trajectory of the stereotactic biopsy

A stereotactic biopsy of the thalamic lesion was performed. Intraoperatively, the specimen was greyish-white, rubbery in consistency, and relatively avascular. Postoperative CT confirmed accurate biopsy trajectory with no hemorrhagic complications (Figure 2). Histopathology revealed a low-grade glial neoplasm with polymorphous morphology. Features included fragments of neuroparenchyma diffusely infiltrated by a glial neoplasm composed of neoplastic glial cells, showing a moderate amount of eosinophilic cytoplasm, and round-to-oval hyperchromatic nuclei with moderate anisonucleosis. Scattered pleomorphic cells were also noted. Mitotic activity was two to three per 10 high-power field (HPF). The stroma showed microcystic changes, increased vascularity, and areas of hemorrhage and calcification. Necrosis and microvascular proliferation were absent. Immunohistochemistry showed diffuse positivity for glial fibrillary acidic protein (GFAP) and CD34. The Ki-67 proliferation index was low. BRAF V600E testing showed positivity (Table 1).

**Table 1:** Immunohistochemistry report Immunohistochemistry findings with features suggestive of a polymorphous low-grade neuroepithelial tumor of the young, CNS WHO grade 1. GAP: Growth-associated protein; OLIG2: Oligodendrocyte transcription factor 2

Immunohistochemistry Report	Results
OLIG2	Positive
Synaptophysin	Positive in few tumor cells
GAP	Diffusely positive
IDH1 p.R132H	Negative
ATRX- retained expression p53	Negative
H3 p.K27M	Negative
H3 p.K27me3	Retained expression
CD34	Highlights ramified neurons and few tumor cells
BRAF p.V600E	Positive
MIB 1 labelling	4%-5%

Postoperatively, the patient remained neurologically stable. The institution's tumour board advised radiation therapy if there is a growth in serial imaging or radical excision in case of neurological symptoms. Follow-up MRI showed no interval change. The patient is currently under follow-up.

## Discussion

PLNTY is a distinct entity within the spectrum of long-term epilepsy-associated tumors (LEAT), sharing overlapping features with dysembryoplastic neuroepithelial tumors (DNET), gangliogliomas, and oligodendrogliomas [1,3,4]. Although the temporal lobe is the most frequent site of origin [3], extratemporal locations, including frontal and occipital lobes, have been described [7,8]. Thalamic involvement, as in our case, is exceptional and has not been previously reported (Table 2) [5,6]. Radiologically, PLNTY appears most often as a non-enhancing, T2 hyperintense lesion with speckled calcification, features that may resemble DNET, ganglioglioma, and oligodendroglioma [9]. However, the absence of cortical involvement and presence of deep gray matter origin in our patient suggested a broader differential, including ganglioglioma and oligodendroglioma. One other confusing element was the minimal contrast enhancement that was seen in our patient. While the WHO description of a classical PLNTY is that of a non-enhancing lesion, authors have reported variable enhancement in their tumors [9].

**Table 2:** Previously reported cases of PLNTY in the literature Previously reported cases of a polymorphous low-grade neuroepithelial tumor of the young (PLNTY) in the literature based on the location [9].

No.	Authors	Year	Ps	Age	Sex	Clinical presentation	Side	Major lobe involvement	Imaging	Contrast enhancement
1	Bale et al. [6]	2021	1	15	F	Seizure	Left	Temporal	Calcified, cystic	Yes
2	Chen et al. [4]	2020	3	14	F	Seizure	Left	Temporal	Calcified, cystic	No
				15	M	Seizure	Right	Temporal	Calcified, cystic	Yes
				16	M	Seizure	Right	Frontal	Calcified, cystic	Yes
3	Gupta et al. [8]	2019	1	30	M	Epilepsy	Right	Temporal	Solid	No
4	Huse et al. [1]	2016	10	16	M	Seizure	Right	Temporal	Calcified	Yes
				18	F	Seizure	Right	Temporal	Calcified, cystic	No
				23	F	Seizure	Right	Temporal	Calcified	No
				17	F	Seizure	Right	Temporal	Calcified	No
				4	M	Seizure	Left	Temporal	Calcified	Yes
				9	M	Seizure	Right	Frontal	Cystic	No
				10	M	Headache	Right	Occipital	Calcified	No
				23	F	Seizure	Right	Temporal	Calcified	No
				32	F	Seizure	Right	Temporal	Calcified	No
				24	F	Visual disturbances	Right	Occipital	Calcified	No
5	Jhonson et al. [10]	2019	9	12	F	-	Left	Temporal	Calcified, cystic	No
				12	F	-	Left	Temporal	Calcified, cystic	No
				26	F	-	Left	Temporal	Calcified, cystic	Yes
				16	F	-	Right	Temporal	Calcified, cystic	No
				25	M	-	Right	Temporal	Calcified	No
				15	F	-	Left	Temporal	Calcified, cystic	No
				5	F	-	Right	Parietal	Calcified, cystic	No
				34	M	-	Right	Temporal	Calcified, cystic	Yes
				17	F	-	Mediam	Third ventricle	Calcified, cystic	Yes
6	Bitar et al. [11]	2018	1	31	M	Epilepsy	Right	Temporal	-	-
7	Riva et al. [12]	2018	1	57	M	Headache	Right	Frontal	Cystic	No
8	Sumdani et al. [13]	2019	1	19	M	Seizure	Right	Parietal	Calcified	No
9	Surrey et al. [14]	2019	6	7	M	Epilepsy	-	Temporal	Cystic	-
				10	F	Epilepsy	-	Parietal	-	-
				14	M	Epilepsy	-	Parietal	-	-
				16	M	Epilepsy	-	Temporal	-	-
				8	M	Epilepsy	-	Temporal	Cystic	-
				14	F	Epilepsy	-	Temporal	Cystic	-
10	Tateishi et al. [15]	2020	1	14	M	Epilepsy	Left	Temporal	Calcified, cystic	No
11	Fei et al. [3]	2022	8	5	M	Epilepsy	Right	Occipital	Cystic	No
				25	M	Seizure	Right	Temporal	Calcified	No
				21	M	Epilepsy	Right	Temporal	Calcified, cystic	Yes
				51	M	Epilepsy	Left	Frontal	Calcified	Yes
				30	F	Seizure	Right	Frontal	Calcified	No
				46	M	Epilepsy	Left	Temporal	Calcified, cystic	No
				28	M	Epilepsy	Left	Temporal	Calcified	No
				47	M	Epilepsy	Right	Frontal	Cystic	Yes
12	Lelotte et al. [16]	2019	1	33	F	Seizure	Right	Temporal	Cystic	No
13	Broggi et al. [17]	2021	1	50	F	Epilepsy	Left	Temporal	Calcified	No
14	Benson et al. [18]	2021	1	44	F	Mood disorder	Left	Temporal	Calcified, cystic	Yes
15	Gupta et al. [19]	2021	5	11	M	Epilepsy	Right	Frontal	-	-
				17	M	Epilepsy	Left	Temporal	-	-
				10	F	Epilepsy	Left	Temporal	-	-
				38	M	Seizure	Right	Temporal	-	-
				11	F	Epilepsy	Right	Temporal	-	-
16	Our case	2022	1	45	M	Seizure	Right	Parietal	Calcified	Yes

Histologically, PLNTY exhibits polymorphous glial architecture with oligodendroglioma-like areas, perivascular rosettes, calcification, and diffuse CD34 positivity [1,4,10]. The diagnosis often hinges on histopathological and immunohistochemical correlation. PLNTY lacks IDH mutations and 1p/19q codeletion, distinguishing it from classic oligodendrogliomas [1,2]. Ganglioglioma is characterized by the presence of true, well-formed ganglion cells with neuronal marker positivity (e.g., synaptophysin and MAP-2).

The pathogenesis involves activation of the MAP kinase pathway, frequently due to BRAF V600E mutations or FGFR2/3 fusions [1,4]. In our patient, the BRAF positivity indicated a diagnosis of PLNTY.

Our patient was asymptomatic other than paresthesias in the limbs. The risk of surgery, we felt, was much more than the benefit of attempting a radical excision. Hence, a stereotactic biopsy was performed for this patient [9].

## Conclusions

This case underscores the importance of considering PLNTY in the differential diagnosis of low-grade thalamic lesions, irrespective of the clinical presentation. Histological confirmation through biopsy remains essential, especially in atypical anatomical locations. Further reports are needed to define the full radiological and molecular spectrum of PLNTY and to guide long-term management in non-epileptogenic cases.
